# Nasal Epithelial Cell-Based Models for Individualized Study in Cystic Fibrosis

**DOI:** 10.3390/ijms22094448

**Published:** 2021-04-24

**Authors:** Duncan E. Keegan, John J. Brewington

**Affiliations:** 1 Division of Pulmonary Medicine, Cincinnati Children’s Hospital Medical Center (CCHMC), 3333 Burnet Avenue, Cincinnati, OH 45229, USA; duncan.keegan@cchmc.org; 2 Department of Pediatrics, University of Cincinnati College of Medicine (UC-COM), 3230 Eden Avenue, Cincinnati, OH 45267, USA

**Keywords:** cystic fibrosis, CFTR, nasal cell, theratyping, personalized medicine

## Abstract

The emergence of highly effective CFTR modulator therapy has led to significant improvements in health care for most patients with cystic fibrosis (CF). For some, however, these therapies remain inaccessible due to the rarity of their individual *CFTR* variants, or due to a lack of biologic activity of the available therapies for certain variants. One proposed method of addressing this gap is the use of primary human cell-based models, which allow preclinical therapeutic testing and physiologic assessment of relevant tissue at the individual level. Nasal cells represent one such tissue source and have emerged as a powerful model for individual disease study. The ex vivo culture of nasal cells has evolved over time, and modern nasal cell models are beginning to be utilized to predict patient outcomes. This review will discuss both historical and current state-of-the art use of nasal cells for study in CF, with a particular focus on the use of such models to inform personalized patient care.

## 1. Introduction

Cystic fibrosis (CF) is a multisystem, autosomal recessive disease characterized by progressive obstructive respiratory failure [1]. Mutations in the gene encoding the Cystic Fibrosis Transmembrane conductance Regulator (CFTR) protein cause CF, with >2000 variants described to date, though not all variants result in CF [2,3]. This protein acts as an ion channel at the epithelial surface, responsible for gated passage of chloride and bicarbonate [4,5,6]. Variants in the *CFTR* gene result in several forms of protein dysfunction or deficiency. For example, the most common variant, F508del *CFTR*, results primarily in improper protein trafficking to the cell surface, which is necessary for protein function [7]. Other variants may result in reduced protein production, altered channel conductance properties, impaired channel gating, or more. Some of these variants have been studied and grouped into classification schemes of the resultant protein problem, though the impacts of many *CFTR* variants remain unknown [8].

Understanding the protein impact of common *CFTR* variants required concerted efforts across many groups and was largely informed by studies of heterologous models over-expressing a single mutant allele [9,10,11,12,13]. These efforts have paid dividends, leading to the creation of small-molecule therapeutics that improve various forms of protein dysfunction [14,15,16,17,18]. Termed “CFTR Modulators”, these drugs have altered the landscape of CF care, shifting from a model of symptom management to one of symptom prevention, treating the protein-level cause of the disease. Highly effective modulator therapies are now available to >85% of patients in North America with CF, and in Phase 3 trials have produced significant improvements in lung function, nutritional status, and patient-reported outcome measures [14,15].

Despite these remarkable accomplishments, several key issues remain. First, modulator therapy is not available to all patients with CF. Some carry two alleles that will not be amenable to small-molecule correction, such as those with two nonsense variants. Others, however, carry alleles that may respond to such therapies, but have not been evaluated due to the rarity of the variant. It has been estimated that approximately half of the >2000 identified *CFTR* variants are carried by 5 individuals in the world or less; these individuals have been, by nature of their rarity, excluded from both clinical trials and heterologous model-driven studies of CFTR biology [10,19,20]. It is particularly noteworthy that such rare and ultra-rare variants are more likely to be present in individuals in minority racial groups, further exacerbating existing disparities in care compared to their Caucasian counterparts [21]. A second issue with the current therapeutic environment is the limited understanding of phenotypic and therapeutic variance in CF. Though modulators are prescribed based upon genotype, prior studies have demonstrated that >50% of disease variance is unrelated to this genetic factor [22]. This disease variance has been re-demonstrated across modulator trials, with wide variation in patient outcomes [14,15,16,17,18].

One proposed way to address these issues is through the study of patient-derived laboratory models generated from disease-relevant tissue sources. Through analysis of patient-specific laboratory outcomes and linked clinical data, the cellular mechanisms underlying disease and therapeutic variance may be elucidated. Using the same models, analysis of CFTR function with various treatments, such as modulators, may provide a preclinical assessment of patient response, and therefore be a useful tool for matching patients with the optimal treatments. This patient-treatment matching has been termed “theratyping,” or classifying a patient’s *CFTR* variants by the efficacy of treatments for those variants [23]. Some have approached such theratyping work in heterologous models, including Fisher Rat Thyroid cells; this approach has been successful in pushing FDA expansion of modulator label indications [24]. This approach, while powerful, is limited to more common variants, and the heterologous model data has not always held true to the human condition [25,26,27]. Using patient-derived models for theratyping efforts, conversely, will theoretically be of greater relevance to the individual subject, representing not only both of their *CFTR* alleles, but also any other genetic or cellular determinants of disease. To be clear, both heterologous and patient-derived models have important roles, strengths, and weakness with relation to optimizing and expanding CFTR-focused therapeutics. For the present review, however, focus will be placed on patient-specific models.

Use of patient-specific models to study CF is not a novel concept. Bronchial epithelial cells grown at air–liquid interface (ALI), for example, have been instrumental in constructing the current understanding of CFTR biology in the airway [28]. These models, however, are highly invasive to acquire (requiring lung transplant), and therefore are not well-suited to patient-specific study [29]. Seeking less invasive sources, many investigators have capitalized on other disease-relevant tissues, with extensive research in intestinal and nasal cell-based models [30,31,32,33]. Intestinal models are frequently used across Europe with great success, and have demonstrated robust growth capacity, clear disease relevance, and promise as a platform for therapeutic development [33]. These models, however, have not gained as much traction in North America, with a limited number of centers performing these assays regularly [23]. Additionally, while the intestine is a critically important tissue in CF disease, the mortality of the disease stems from the respiratory tract. As ion transport in the airway and the gut are not equivalent, a respiratory model may carry benefits over an intestinal source.

In this review, we will focus on primary human nasal epithelial (HNE) cell culture as a patient-specific laboratory model of CF. Particular attention will be paid to frequently used models, including ALI and organoid cultures, discussing strengths and weakness of each. The relevance of these models to lower airway models will be assessed, as well as to the clinical disease state itself. Finally, consideration will be made for the future needs to maximize and validate HNE models as a method for translational study, including theratyping efforts.

### 1.1. Historical Nasal Cell Models

Conceptually, the use of nasal models to understand the functional role of ion transport in CF may have started with the description of functional, in vivo differences in nasal potential difference between CF and non-CF subjects in 1981 [34]. Subsequent studies using this assay demonstrated disease-relevant properties of the nasal mucosa, such as increased sodium transport and decreased chloride efflux in subjects with CF, which had been previously described in difficult-to-obtain lower airway samples [35]. Transitioning these in vivo studies to in vitro assays to better understand the cellular mechanisms of such observations was a logical step, but impeded by a lack of reliable culture methodology. One early method, for example, was the repopulated heterologous graft technique, in which human nasal cells were dissociated from polyp samples, seeded onto denuded murine tracheal explants, and then implanted into immune-deficient mice [36]. After several weeks, these tracheal implants were collected and assayed for ion flux. This system generated useful results, but viability and repeatability were limited, with early reports of culture success well under 50% [36].

A simpler system for culturing nasal cells became more popular in the mid-1980s, relying on suspension culture using serum-supplemented media. An early description of this technique demonstrated excellent success rates for short-term cultures of polyp tissue, and utilized this model to characterize deficient chloride uptake in these nasal cells [37]. Importantly, however, phenotypic analysis of the cultured cells did not reveal a classic respiratory morphology, lacking the columnar shape and organization seen in non-dissociated samples [37]. Nonetheless, these submerged, incompletely differentiated cultures became quite useful in describing the physiologic impact of CFTR deficiency on the airway epithelium. Through the use of chloride uptake/efflux assays, patch-clamp, and more, these studies demonstrated altered ion transport, increased transmembrane potential difference, increased sodium permeability, and reduced chloride permeability in samples from those with CF, all now hallmarks of the CF respiratory epithelium [37,38,39,40,41]. Similar cultures were utilized through the late 1990s to study respiratory ciliogenesis, CF-relevant host-pathogen interactions (e.g., *Pseudomonas aeruginosa*, *Staphylococcus aureus*), gene therapy, and early F508del protein correction with therapeutics [42,43,44,45,46,47,48].

In this submerged culture system, two key limitations reduced both throughput and applicability. Submerged cultures of nasal cells did not fully recapitulate the mature respiratory epithelium, with impaired morphologic differentiation [37]. This lack of differentiation may reduce the in vivo disease relevance, especially in studies where mature tight junction and barrier formation is essential (e.g., gene therapy). The ability to repeat such studies with high numbers was also limited by poor replication of nasal cells in vitro. Unlike other tissue sources with high growth capacity (such as tumor cells), overall expansion of nasal cells using traditional methods is limited to a few passages. In order to overcome this, investigators primarily utilized tissue sources with high cell yield; specifically, polyp and surgical specimens [37,41]. This drastically reduced the potential subject recruitment pool to a convenience sample of those undergoing surgical procedures, largely eliminating pediatric subjects from consideration. Attempts were made at culture using samples obtained by cytology brushing but had limited success. In one such early brushed nasal cell study, while numerous endpoints were achieved, only 7 of 17 cultures reached confluence for full study, highlighting the limited expansion potential of HNEs [49].

A number of methods were trialed to overcome this limitation. Immortalization of HNE cultures was feasible and successful, but required resource commitment, and the long-term relevance of the cultures was unclear [50]. Several modifications of the early culture media, such as altering the serum component or adding feeder fibroblasts, allowed for improved, but still suboptimal expansion [51,52]. Ultimately, however, it was the emergence of conditional reprogramming culture (CRC) technologies in 2012 that allowed for more widespread use of HNEs for CF study [53]. This method utilizes media containing a Rho kinase inhibitor (Y-27632) and irradiated feeder fibroblasts to drastically improve cell growth across a number of tissue sources [53]. In nasal and lower airway cells, the use of CRC methodology has been reported to allow >300-fold expansion of the initial cell sample compared to traditional methods through enriching a progenitor population and preserving the basal cell characteristics of the culture, potentially via a KLF11-dependent action [54,55]. This approach has empowered brushed HNE cell studies from infants up to adults, bypassing the need for surgical samples [56,57].

Novel methods of increasing cell expansion have also emerged, such as dual-SMAD inhibition, but are not yet well characterized [58]. Prior to routine adoption of these methods, additional studies will be required to optimize protocols and define differences and similarities between methods. Regardless, numerous HNE studies now utilize CRC or other expansion methodology to allow for improved cell growth, followed by culture into a differentiated model such as ALI or spheroid cultures. These well-differentiated HNE cultures, patterned after success with bronchial or intestinal models, continue to increase the disease relevance of cultured HNE studies by recapitulating the native airway morphology.

### 1.2. Air–Liquid Interface Cultures

Primary human bronchial epithelial (HBE) tissue planar cultures grown at ALI have been instrumental in therapeutics development for cystic fibrosis and have played an important translational role in leading to the FDA approval of the current modulators. HBE planar cultures remain the gold standard in human tissue-based respiratory models for CF study [23]. The traditional source of tissue for these cultures is explanted lung tissue obtained during lung transplantation and therefore is quite rare, resulting in limited utility for theratyping. The need for a more readily available respiratory tissue source led to the development of HBE cultures from cells obtained during sedated bronchoscopic procedures, and eventually to HNE cultures grown at ALI with cells obtained from nasal brushing [28,59,60]. The application to theratyping is thus drastically improved with minimally invasive tissue collection which even allows for recollection in instances of contamination or failed culture growth.

First described in the late 1980s, culture at ALI allows for the growth of well-differentiated pseudostratified mucociliary epithelium with important physiologic characteristics of in vivo airway epithelium [28,60,61]. In this method, cells are plated onto suspended, semi-porous membranes, with media below the membrane and air above the cells [60]. While prior attempts at primary airway culture grown submerged on plastic dishes resulted in a poorly differentiated squamous morphology, it was found that cellular polarization enabled by the ALI orientation proved critical to mucociliary differentiation [60]. Long-term culture growth over numerous subsequent passages was enabled by the use of fibroblast feeder layers to stimulate proliferation [59].

Electrophysiological assessment of HBE planar cultures grown at ALI was critical in advancing the biologic understanding of CF. In this assay, short circuit current and transepithelial voltage across the polarized membrane is measured in Ussing chambers [62,63]. A series of solutions is applied to the culture to isolate and stimulate CFTR so that ion efflux through CFTR can be directly measured. This technique allows for the detection of CFTR activity and assessment of alterations in function provoked by modulators or other substances, and has been critical in modulator development and expansion [13,24,64,65]. In addition to electrophysiologic assessment, evaluations of ciliary function, inflammatory response, and airway surface liquid physiology have been described using cultures grown in ALI [66,67,68,69].

**Figure 1:**
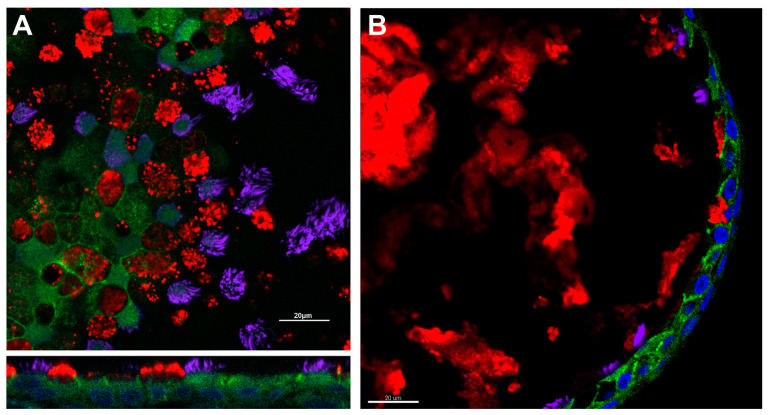
Respiratory epithelial characteristics of HNE models. Immunofluorescence demonstrates the presence of adherens junctions (e-cadherin, green), cilia (acetylated alpha-tubulin, purple), and mucus production (muc5ac, red) in air–liquid interface (Panel (**A**)) and spheroid cultures (Panel (**B**)). Nuclei are highlighted in blue by DAPI. Scale bar = 20 µm. Image courtesy of Brewington laboratory.

To adapt these techniques to nasal cells, brushed and expanded nasal cells are directly seeded onto porous supports. Media, which varies by lab, maintains the culture, promotes differentiation and expansion, and is exchanged regularly [31,70]. Depending on the media used, HNE ALI cultures mature within 4–6 weeks [31,70,71]. The HNE culture recapitulation of lower airway epithelial morphology and physiology allows for HNE use in theratyping as well as in tissue-specific physiologic studies; immunofluorescence demonstrating such morphologic characteristics can be found in Figure 1.

Electrophysiologic assessment of CFTR activity in HNE ALI cultures has the capacity to discriminate between CF, WT, and CF disease with partial CFTR function, and modulator corrected mutant CFTR [72,73]. Among subjects with CF, modulated, ex vivo CFTR activity in HNE cells has been shown to correlate with clinical improvements ppFEV_1_ and sweat chloride, both at the individual level and against historical trial cohort data [32,70,71,72,74,75]. While Ussing chamber studies represent the gold standard in electrophysiological assessment of modulator-rescued CFTR function they are limited by the need for highly specialized equipment and a time and labor intensive process, resulting in a low-throughput assay. A medium-throughput assay for profiling of CFTR modulator efficacy that uses fluorescence-based identification of chloride ion conduction has been described in HNE with good congruence to Ussing chamber data in showing individual patient response to modulators [76].

Despite the rapidly emerging evidence, the use of HNE planar cultures grown at ALI remains limited by uncertainty regarding the relevance of nasal cell culture to the lower airways, specifically in disease states such as CF that have profound lower airways pathology. There is additional uncertainty regarding the relevance of the in vitro environment to the actual human nasal or respiratory epithelium. While the studies above have linked nasal cells to individual or unrelated cohort patient studies, the numbers in these comparisons are small, and the precision of this linkage remains unclear [70,71,72,74,75]. Primary human nasal cell cultures are limited by the number of times they can be passaged as well as by a risk for contamination as compared to immortalized cell lines, though the relevance of primary culture likely offsets this downside for human studies. Notably, cells for nasal tissue culture are more accessible than bronchial or intestinal cells and can be obtained with minimal discomfort to the patient.

### 1.3. Three-Dimensional Cultures

In contrast to ALI cultures, several three-dimensional nasal cell culture models have been described for CF study. Many, though not all, of these models meet the generally held definition of an “organoid,” including three-dimensional structure recapitulating the in vivo tissue coupled with recreation of a tissue-specific function (e.g., mucus production, ciliary motility). Given the relative simplicity of these HNE models, however, most have utilized the term “spheroids” or “nasospheroids” [57,77,78]. For this review, we will utilize the unifying term “spheroids,” but readily acknowledge the laxity of terminology. Regardless of nomenclature, these models all share a three-dimensional, cystic form in which epithelial cells reliably polarize and recreate physiologic characteristics of the respiratory epithelium.

Early descriptions of HNE spheroids from the 1990s largely focus on a submersion culture method. In these models, brushed HNE cells or dissociated nasal polyp tissue is plated in culture flasks or dishes, then manually moved either by hand or on a shaker for the first days of growth [77,79]. This mechanical disruption prevents cell adhesion to the culture vessel, encouraging instead the formation of spherical clusters of epithelial cells with a clear inner lumen, and epithelial cells polarized with the apical surface to the outside. These spheroids morphologically resemble the intact airway epithelium, and provided a model for studies of fluid transport, mucociliary clearance, and ciliogenesis [77,79,80,81,82]. In addition to these physiologic studies, these apex-out, floating spheroids were studied using microelectrodes to characterize ion transport across the nasal mucosa [83]. This study demonstrated clear differences between CF and non-CF samples, establishing the capacity of nasal spheroids to discriminate incremental CFTR function [83].

More recently, investigators have adapted methodology utilized for intestinal organoid studies in CF to nasal spheroids. In this approach, expanded HNEs are embedded in a three-dimensional matrix (typically Matrigel, Corning, NY, USA), which supports the spherical organization of the cells without the need for mechanical agitation [30,57,78,84]. Much like previous spheroid models, these cultures recapitulate the mature respiratory epithelium, including ciliation, mucus production, and formation of cell-to-cell junctions [57,78]. Immunofluorescence demonstrating these morphologic characteristics can be found in Figure 1. By altering the matrix density, cultures can be generated with the epithelial cell apex to the inside/lumen of the spheroid or to the outside [57,78]. To quantify CFTR function, ion transport is stimulated through cAMP-dependent pathways and spheroid size is monitored over time [30,57,78,84]. Using this approach, several groups have reported the capacity of these spheroids to discriminate CF cultures from non-CF, and to demonstrate pharmacologic rescue of CFTR using modulator compounds [57,78,84].

There are several potential benefits to these three-dimensional models over ALI cultures, as well as limitations. After an expansion phase, these cultures reach maturity and readiness for study within 1–2 weeks, typically twice as quickly as ALI cultures [57]. In addition, this methodology can generate hundreds of spheroids, drastically increasing the testing and analysis possibilities over a relatively small number of ALI cultures [30,78]. The analysis of these cultures can be partially automated, increasing throughput [84]. Moreover, by analyzing fluid efflux, these models provide a complimentary readout that may have more direct physiologic relevance to the CF disease state compared to measurements of ion transport in ALI cultures.

Conversely, these spheroid cultures carry limitations relative to ALI cultures. For one, the model variance in spheroids has been reported to be greater than that typically associated with ALI cultures. This is partially offset by increasing the number of measurements but must be considered in study design [57,78]. In addition, the relative novelty of this approach requires robust study to link model results to clinical or established laboratory data before relevance can be established. These efforts are limited by a lack of shared and optimized SOPs, reducing the capacity to compare studies between labs. Due to these limitations, HNE spheroid cultures have, thus far, been less utilized compared to ALI cultures.

### 1.4. Relevance of Nasal Cell Models to the Lower Airways

Establishing the relevance of nasal cell models to the lower airways is of obvious importance to any attempt to use these models for theratyping, personalized studies, or more broadly as surrogates for the study of lower airways diseases. There is a growing body of evidence that suggests that there is sufficient similarity between the morphology and function of nasal cell models and bronchial epithelial tissue to allow HNE to be used broadly as surrogates for HBE in future research in CF and other lower airways disease [31,32,73,85,86]. Several studies have already reported correlations between the results of studies in nasal cell culture models and meaningful clinical outcomes [70,71,72,74,75]. However, it is important to note that there may be specific situations in which HNE cultures would not be suitable for use as a surrogate for bronchial epithelium and the criteria to determine their suitability is ultimately contingent upon the exact question being asked.

Characterization of the structure, morphology, and cellular composition of HNE planar cultures has demonstrated the recapitulation of specific characteristics of mature respiratory epithelia [31,66]. Submerged nasal epithelial cultures are visually indistinguishable in their morphology from submerged bronchial epithelial cultures, have similar receptor expression and, although they differ in absolute mediator levels, both show similar response to cytokine stimulation [85]. Submerged cell culture models, however, are undifferentiated and have mostly been supplanted by cultures grown at ALI as they appear to have greater fidelity to in vivo conditions. HNE cultures grown at ALI display pseudostratified columnar epithelial morphology with a cellular composition that consists of ciliated cells, mucous producing cells, basal cells, and ionocytes [31,66,87]. The number of ionocytes in nasal epithelial cultures and bronchial cultures is significantly different although CFTR function is not, and it is thought that the difference reflects a proximal to distal gradient in ionocyte numbers within the respiratory tract [87]. The presence of tight-junctional proteins has been confirmed in multiple studies and contributes to epithelial barrier integrity within HNE planar cultures at ALI [31,66].

Physiologically, HNE cultures have been shown to recapitulate key components of HBE cultures that are critical to the analysis of airways diseases. For example, electron microscopic examination has shown that the cilia in fully differentiated HNE planar cultures grown at ALI have a typical ultrastructure with normal axonemal microtubule and dynein arm arrangement as well as intact ciliary beat [66]. This allows for the evaluation of ciliopathies in the absence of possible confounding “secondary” dysfunction of cilia that can occur in fresh primary cells upon harvesting [67]. Of particular interest in CF, electrophysiologic assays have demonstrated the presence of functional CFTR in HNE cultures, and paired samples of brushed HNE and brushed HBE from the same patient (among a cohort of patients with CF) have shown similar CFTR dependent ion transport and a strong correlation in the ability to detect CFTR response to modulation between the two culture types [31].

In other disease states direct comparisons between HNE and HBE cultures have supported the use of HNE cultures as surrogates for HBE cultures with some limitations. In asthmatic patients the physiologic response to IL-13 stimulus is similar between HNE and HBE cultures at ALI, but there are distinct morphologic differences with respect to the number of goblet and ciliated cells in the nasal epithelial cultures compared to the bronchial epithelial cultures [88]. This may reflect an inherent restriction of goblet cell hyperplasia to the lower airways in asthma but is an important consideration in terms of model selection. Nasal cell cultures have been used in the study of viral entry, receptors and internalization, viral replication and innate immune responses [89]. Studies using paired nasal and bronchial cell cultures have shown similar antiviral and viral induced pro-inflammatory responses between to the two tissue sources [90]. Nasal gene expression profiling has the ability to differentiate patients with COPD from patients without COPD, and analysis of the nasal versus bronchial COPD gene expression profile shows significant overlap between the epithelia [91]. Comparisons of the inflammatory response in paired HNE and HBE cultures at ALI from patients with COPD shows correlation in IL-8 response to pseudomonas aeruginosa lipopolysaccharide between the culture types but differing IL-6 response, as well as differential response to cigarette spoke exposure in Toll-like receptor 4 expression [92].

Taken together, these studies demonstrate overall similarities in structural and functional characteristics of HNE and HBE cultures at ALI, though subtle differences exist. While differences in inflammatory mediators or genomic profiling may prompt hesitancy for certain investigations, functional analyses of CFTR activity have consistently supported HNEs as an adequate surrogate for HBE study [30,70,71].

### 1.5. Utility of HNE Models for CF Therapeutics Analysis

The development and approval of CFTR modulator compounds have dramatically altered the landscape of CF care. Ivacaftor, the first of these compounds to be approved, is a “potentiator,” directly increasing the open probability of CFTR protein at the cell surface, thereby increasing chloride and bicarbonate membrane permeability [24]. This drug is effective as monotherapy for a number of variants with protein defects in channel gating or conductance [12,17,93,94]. However, to correct protein defects that alter folding and trafficking, additional compounds are required. Termed “correctors,” these drugs improve the intracellular processing of the translational end products of mutated *CFTR* resulting in a net increase in the amount of protein that is successfully trafficked to the cell surface, where channel activity can subsequently be modulated by a potentiator [13]. Three corrector compounds are currently FDA approved for use: lumacaftor (combined with ivacaftor as Orkambi©), tezacaftor (combined with ivacaftor as Symdeko©), and elexacaftor (combined with tezacaftor and ivacaftor as Trikafta^TM^) [14,15,16,18,64]. All three were developed to target F508del *CFTR*, but have been shown effective in other groups as well [95]. Elexacaftor, tezacaftor, and ivacaftor (ETI) therapy has specifically produced robust clinical responses in a large proportion of the CF population, including F508del homozygotes, heterozygotes, and select rare variants identified through heterologous screening assays [14,15,64]. Numerous additional corrector and potentiator compounds have been identified, and are in development as possible therapeutics [96,97,98]. In addition, other classes of modulator compounds are under study, including those that increase mRNA signal (“amplifiers”), though none of these therapies are currently FDA approved [99,100].

In considering optimal modeling of modulator activity, relevance of the cellular context to the in vivo condition is necessary. This may be less important for analysis of potentiator compounds, where compounds bind directly to protein already at the cell surface, but is critical in considering the action of corrector compounds, which are dependent upon the cell-specific processing for action [101]. The processes by which the current correctors improve protein folding and trafficking to the cell surface are not clearly delineated and are likely much more dependent on the intracellular and tissue level milieu, therefore increasing the importance of high fidelity model systems that accurately recapitulate in vivo conditions [102]. The use of HNE models to meet this need has been instrumental in evaluating the therapeutic potential of small-molecule modulators, as well as in helping to understand the mechanism of action of these molecules and enable the identification of susceptible mutations [65,95,96,97,103]. Nasal cell testing, along with other model systems, has demonstrated the action of elexacaftor as a type 3 corrector compound with efficacy in numerous CFTR variants beyond F508del such as G85E and M1101K *CFTR* [96]. Indeed, as more modulator therapies are identified, nasal cell models are increasingly useful to quantify response in common and rare *CFTR* variants, which may aid drug development and approval [97,103]. Patient-derived nasal epithelial models have allowed for flexibility in testing therapeutic response, showing specific promise as a validation tool following profile screening by higher-throughput methodology, gene edited cells, or immortalized cell lines [104]. Amplifiers have also been tested in combination with other modulators for rare mutations using HNE model systems [104]. Additional work is ongoing to adapt such HNE protocols for standardized, optimized characterization of CFTR correction [65].

In addition to preclinical development and drug characterization, HNE models have been utilized in the personalized evaluation of rare-mutant specific response to CFTR modulators. In this approach, patients with rare mutations that were not originally approved for modulator treatment have undergone nasal cell culture for ex vivo evaluation of modulator responsiveness [70,71,75,105,106]. Though currently in small numbers, reports of using HNE-based data to procure insurance coverage for biologically active modulator drugs are increasing [71,75,107]. Importantly, these HNE data have shown correlation to patient outcomes, validating this theratyping approach [70,71,74,75]. Using these techniques, theratyping has begun to fill the gaps left by large, randomized control trials. Surveys demonstrate patient and family acceptance of the use of ex vivo tissue for theratyping and show a preference for the nose over the rectum or lung as source tissue [108]. Theratyping represents a leading edge of precision-targeted therapeutics for those with rare variants, an underserved subset of the CF population, and may be a key tool in the goal of bringing highly effective therapies to all persons with CF.

Potential therapeutic agents directed at the underlying cause of disease in CF are not limited to small-molecule modulator agents. A particularly difficult group of *CFTR* variants to correct are those with a premature termination codon (e.g., G542X), which do not produce a full-length protein and are insensitive to the available modulator compounds. Extensive work is underway to identify compounds to facilitate read-through of the aberrant stop codon, or to reduce nonsense mediated decay of the abnormal mRNA. HNEs have been used to quantify nonsense mediated decay across several such variants, demonstrating capacity as a model for corrective therapies [109]. Similarly, HNEs have served as part of a platform analyzing read-through agents such as gentamicin [110].

Finally, therapies that are independent of the underlying *CFTR* genotype offer the potential for broad and impactful changes in the entire CF community. Nasal cell culture may be utilized in similar way in the development of such treatments. For example, nasal cells have also been utilized to study therapies targeting other ion transporters, such as TMEM16A, which may hold promise as a *CFTR* genotype-agnostic therapy [111]. There are concerted efforts to create practical and effective genetic treatments for CF, including gene therapies and gene editing. This area has seen significant growth with the development of novel tools such as CRISPR/Cas, though highly effective therapies remain elusive at this time. While traditional studies in developing gene therapy have utilized bronchial epithelial cells, the potential for transition to an HNE platform is logical and is currently being utilized [112,113]. In addition to providing an accessible platform for preclinical study, HNE culture work easily translates into early clinical trials, where nasal cells could be used to optimize delivery and to measure efficacy after treatment, either in culture or through the nasal potential difference assay [114]. As the field moves forward in developing these promising therapies, HNE assays may offer a powerful tool in creation, analysis, and fine-tuning of treatments.

## 2. Discussion

The phenotypic variability in cystic fibrosis, the diversity of *CFTR* variants, and the life-changing promise of modulator therapy make cystic fibrosis a perfect disease for the application of personalized medicine. While the current genotype-directed approach to personalized care is powerful, key gaps in drug access and the understanding of therapeutic variance remain. Through the use of patient-derived, tissue-based models for personalized study and theratyping, these gaps may begin to close. As such, the use of nasal cell models to evaluate an individual’s genetic variants for response to modulator can serve as a pathway for modulator therapy to reach individuals with rare variants.

Nasal cell models carry key benefits over traditional methods, including personalization of the assay and perhaps increased relevance to the in vivo condition [25]. Numerous studies have demonstrated similar model characteristics between HNE and HBE cultures, which have largely been considered a gold standard model. Moreover, emerging evidence supports the relevance of both ALI and organoid models based on HNE culture to the clinical outcomes of the individual patient. Nonetheless, key barriers remain to the full realization of these techniques.

First, there is a pressing need to streamline and standardize processes across groups. Published data on models that appear very similar at first glance oftentimes reveal significant differences in results. One of the key differences is variance in the magnitude of CFTR functional measurements in common groups (e.g., healthy controls subjects), which limits the ability to compare data from different labs [31,70]. In the absence of consensus on optimal growth, media composition, and expansion methods, these differences will continue [58]. Notably, media selection appears to drive variability in epithelial differentiation which is particularly significant given the lack of standardization in culture expansion and maintenance techniques and the lack of uniformity in evaluating and verifying differentiation [115,116]. While it is possible to normalize functional measurements to within-lab data in healthy control subjects (e.g., as a percent of “normal” CFTR function), this numerical normalization ignores what may be significant differences in the models (e.g., differentiation and cell types). Much focus is needed in this area to allow for the widespread adoption of common methodology.

Secondly, while these HNE-based models have demonstrated relevance to the in vivo condition, as noted above, the precision of the correlation of these in vitro measurements to meaningful clinical outcomes remains unknown. Most comparisons of laboratory and clinical data are small in number, or are compared against historical cohorts instead of the source individuals [32,70,71,72,74,75]. As such, it remains unclear how uniquely these models represent an individual subject, and how well data from such models can be carried into clinical care. This is particularly relevant for theratyping efforts in CF, where model predictions may be translated directly into therapies [71]. Additionally, the threshold used to define a clinically relevant response in these models remains unclear, limiting their application to theratyping.

Finally, the present use of HNE models for theratyping is limited to those subjects carrying rare, missense *CFTR* variants. For the cohort of patients with nonsense mutations that result in truncation of protein that is not amenable to correction with the currently available modulators, the presently described assays and process of theratyping may not be of use. HNE models, however, hold promise for adaptation to the development and analysis of next generation therapeutic agents. For example, nasal cells could be used both as a development model for a gene editing or gene therapy approach, as well as an early-phase readout for clinical trials of that therapy or other novel therapies [106,113,117,118,119]. Similar approaches focused on nasal potential difference have already been used with antisense oligonucleotide therapy, and could easily be adapted to culture-based models [114]. In this way, HNE-based models hold potential for the adaptation to those with nonsense mutations, as well as to other lines of personalized study in the future.

Human nasal epithelial cultures are a powerful tool for the study of respiratory disease, and are emerging as a key facilitator of personalized study and care in CF. Whether cultured at ALI or grown as organoids, these models demonstrate the capacity to quantify CFTR function and modulation, as well as to recapitulate key structural and functional components of the lower airway disease associated with CF. While much work remains to be done in optimizing and generating consensus among protocols, great promise exists for the translation of these models into clinical care to maximize the benefits of available and emerging therapies in CF.
